# The prevention of fragility fractures in patients with non-metastatic prostate cancer: a position statement by the international osteoporosis foundation

**DOI:** 10.18632/oncotarget.17980

**Published:** 2017-05-18

**Authors:** Luisella Cianferotti, Francesco Bertoldo, Marco Carini, John A. Kanis, Alberto Lapini, Nicola Longo, Giuseppe Martorana, Vincenzo Mirone, Jean-Yves Reginster, Rene Rizzoli, Maria Luisa Brandi

**Affiliations:** ^1^ Department of Surgery and Translational Medicine, University of Florence, University Hospital of Florence, Florence, Italy; ^2^ Department of Medicine, University of Verona, Verona, Italy; ^3^ Department of Urology, University of Florence, University Hospital of Florence, Largo Brambilla Florence, Italy; ^4^ Centre for Metabolic Bone Diseases, University of Sheffield, Sheffield, United Kingdom; ^5^ Department of Urology, University of Naples Federico II, Naples, Italy; ^6^ Department of Urology, S Orsola-Malpighi Hospital, University of Bologna, Bologna, Italy; ^7^ Department of Public Health, Epidemiology and Health Economics, University of Liege, CHU Sart-Tilman, Liege, Belgium; ^8^ Service of Bone Diseases, Geneva University Hospitals and Faculty of Medicine, Geneva, Switzerland

**Keywords:** osteoporosis, androgen deprivation therapy, ADT, FRAX, zoledronic acid

## Abstract

Androgen deprivation therapy is commonly employed for the treatment of non-metastatic prostate cancer as primary or adjuvant treatment. The skeleton is greatly compromised in men with prostate cancer during androgen deprivation therapy because of the lack of androgens and estrogens, which are trophic factors for bone. Men receiving androgen deprivation therapy sustain variable degrees of bone loss with an increased risk of fragility fractures. Several bone antiresorptive agents have been tested in randomized controlled trials in these patients. Oral bisphosphonates, such as alendronate and risedronate, and intravenous bisphosphonates, such as pamidronate and zoledronic acid, have been shown to increase bone density and decrease the risk of fractures in men receiving androgen deprivation therapy. Denosumab, a fully monoclonal antibody that inhibits osteoclastic-mediated bone resorption, is also effective in increasing bone mineral density and reducing fracture rates in these patients. The assessment of fracture risk, T-score and/or the evaluation of prevalent fragility fractures are mandatory for the selection of patients who will benefit from antiresorptive therapy. In the future, new agents modulating bone turnover and skeletal muscle metabolism will be available for testing in these subjects.

## INTRODUCTION

Prostate cancer is the fourth most common cancer and the most frequently diagnosed malignancy in men. In 2013, there were 1.4 million new cases of prostate cancer and 293,000 prostate cancer-related deaths [1], with 57% of cases occurring in developed countries, and a 5-year prevalence of 3,858,000 cases (data source: http://globocan.iarc.fr/Pages/fact_sheets_cancer.aspx). It is estimated that 1 in 7 men will be diagnosed with prostate cancer during his lifetime. In Europe, 416.7/100,000 new cases of prostate cancer were diagnosed in 2012, with rates increasing continuously [2, 3]. Besides being a reflection of population aging, much of the increase in the incidence worldwide can be ascribed to prostate-specific antigen (PSA) testing and incident detection of prostate cancers following trans-uretral resection of the prostate.

Since the introduction of testing for PSA in the ‘90s, a large majority of patients with prostate cancer have been diagnosed at early clinical stage [4]. Nonetheless, despite the high frequency of low-risk tumors, locally advanced and metastatic tumors are still identified at diagnosis [5]. Only 40% of patients with high-risk localized tumors treated with radical prostatectomy and/or radiotherapy remain free of cancer recurrence at 10 years. In these cases, additional surgical or chemical-medical hormone deprivation therapy is strongly recommended. Regarding metastatic disease, as pointed out by systematic analyses of the secular trend of this disease, the pattern of metastasis has gradually changed in recent decades, with a decreased rate of osseous metastases and increased frequency of non-osseous ones [6].

Despite the decreasing incidence of advanced prostate cancer, studies on prescription databases have shown that the use of ADT is increasing since it is now frequently employed, outside the guidelines, as an adjuvant treatment in early, non-metastatic prostate cancer [7]. Moreover, when established early after first-line management, this therapy can last for decades [7], putting these patients at an increased risk for fragility fractures in the long term.

Anti-hormonal therapy is associated with increased survival rates in prostate cancer patients. However, it is also the reason for long-term side effects, often complicated by concomitant comorbidities [5, 8–10]. Recently, an association with ADT and increased non-cancer mortality in older patients with non-metastatic prostate cancer has been shown [11].

Cancer-associated bone disease can be the result of cancer itself (i.e. malignancy-dependent bone involvement) due to tumor-derived circulating bone resorbing molecules or metastases and/or anti-hormonal therapies against the primary disease (i.e. cancer treatment-dependent bone involvement) [12]. In prostate cancer, bone is the main target organ for osteoblastic and osteolytic metastases, often giving rise to pathologic fractures due to local effects [12]. At the same time, anti-hormonal treatment is a major cause of bone loss with an increased risk for fragility fractures, hampering the quality of life of patients and increasing mortality [13, 14].

The main drugs proven to be effective in postmenopausal and senile osteoporosis have been shown to be effective in primary and secondary prevention of osteoporosis in prostate cancer, and to decrease the risk of fragility fractures [15].

In February 2015, representatives of the International Osteoporosis Foundation (IOF) and leading experts in the field of prostate cancer convened at a meeting in Florence (Italy) to present and discuss the existing information on this subject.

The aim of this document is to specifically address the available evidence on cancer-treatment–dependent bone disease in non-metastatic prostate cancer and evidence on the efficacy and effectiveness of antiosteoporotic treatments in these patients, coming from observational, interventional and expert consensus, in order to give guidance on this subject. Indeed, several scientific societies and working groups have approached this argument in the recent past, attempting to draw up some guidelines on the monitoring and treatment of secondary osteoporosis in cancer patients, although no papers were specifically focused on male subjects with bone loss secondary to androgen-deprivation therapy (ADT) for hormone-sensitive prostate cancer [16]. Given these considerations, the management and prevention of bone metastases in prostate cancer patients, which have been fully addressed in recent publications [12], have intentionally been excluded from this paper.

## MATERIALS AND METHODS

A review of the available literature has been carried out. Papers were retrieved by means of a PubMed enquiry (up to November 2015) using the following terms: “prostate cancer”, “epidemiology”, “androgen deprivation therapy” and/or “adjuvant therapy” AND “osteoporosis”, “fractures”, bone fragility”, “bone remodeling”, “bone turnover”, BMD, “skeletal homeostasis”, “bone metabolism”, “fracture risk assessment”, FRAX”, “muscle”, “sarcopenia”, “antiresorptives”, “bisphosphonates”, or active principle of bisphosphonates (such as pamidronate, alendronate, risedronate, zoledronate), “denosumab”, “calcium”, “vitamin D”, “cost effectiveness analysis”. International guidelines from Cancer and Bone Societies and have been taken into account and integrated. Data were retrieved, summarized, and incorporated in order to provide an objective, complete description of the available evidence. Finally, main review articles on the subject were retrieved and appropriately cited.

### Androgen deprivation therapy (ADT) in prostate cancer

Prostate cells are physiological targets for androgens, which stimulate their function, growth, and proliferation [17]. Indeed, testosterone, although not tumorigenic itself, promotes the growth, proliferation and propagation of androgen-sensitive tumor cells. Conversely, prostate cells deprived of androgenic stimulus undergo apoptosis [18]. ADT refers to any treatment eventually resulting in the suppression of androgen activity [19]. ADT can be attained both by suppressing the secretion of testicular androgens (by means of bilateral orchiectomy or long acting GnRH agonists or antagonists) or by inhibiting the action of circulating androgens at the level of their receptor using competing compounds (by means of non-steroidal antiandrogens or steroidal antiandrogens). In addition, these two therapeutic approaches can be used together to reach the complete, also referred to as maximal or total, androgen blockade (CAB) [20].

Surgical castration, the best option to realize ADT, leads to a substantial decrease of testosterone levels, i.e. below 20 ng/dl (or 1 nmol/L). However, the definition of castration level obtained with medical ADT and considered by regulatory authorities for disease control is a serum testosterone below 50 ng/dl (or 1.7 nmol/L) [20].

Long-acting LHRH agonists (such as goserelin, leuprolide, triptorelin) are currently the most commonly employed drugs in ADT. Chronic exposure to LHRH agonists results in the desensitization of LHRH-receptors, suppressing LH and FSH secretion and, consequently, testosterone production. Castration level is usually obtained within 2–4 weeks. LHRH antagonists, such as degarelix, reduce testosterone levels more rapidly and do not cause flare-up phenomena, as LHRH agonists do at the beginning of therapy due to transient stimulation of FSH and LH and consequent testosterone surge. These compounds can be administered either alone or in combination with androgen receptor antagonists, such as flutamide, nilutamide, or bucalutamide, in order to reach CAB [21]. Two additional compounds, abiraterone acetate and enzalutamide, targeting the androgen axis, have recently been developed in order to blunt the effects of intracellular androgens, which are increased in castration-resistant prostate cancer [22].

ADT is considered the gold standard therapy for locally advanced or metastatic androgen-dependent prostate cancer in order to support long-term benefits, according to several international guidelines [20, 21].

There are many issues around anti-hormonal therapy, such as treatment of early stage prostate cancer, the best time to start (immediate *vs* deferred), and the best means of administration (partial *vs* maximal blockade, intermittent *vs* continuous). Indeed, adverse effects of life-long androgen deprivation may be avoided in a substantial number of patients with a deferred treatment policy [5].

To date, ADT is indicated for symptomatic patients with metastatic disease, or extensive T3-T4 histological staging, or high PSA level (> 50 ng/ml) or (> 25 ng/mL and PSA doubling time < 1 year), and in the case of at least 2 positive lymph nodes after extended lymph node dissection [21, 23]. Conversely, ADT is not usually advised for early stage prostate cancer due to a lack of evidence of long-term benefits [4].

ADT has also been employed as adjuvant therapy in place of radiotherapy before prostatectomy in the case of locally advanced tumors with negative lymph nodes, in asymptomatic patients with metastasis, or in men with localized prostate cancer who are unfit for surgery or radiation. However, in these latter cases, no sufficient evidence deriving from properly conducted randomized controlled trials (RCTs) for a beneficial ADT-mediated effect on disease-free survival, local disease control, or mortality rate, has been demonstrated with respect to non-active surveillance or other therapies [20, 21]. Continued testicular androgen suppression with LHRH analogues in CRPC is debatable. However, in the absence of prospective data, the modest potential benefits of a continuing castration outweigh the minimal risk of treatment [21, 24].

Once medical ADT is established, soon after first-line management, it can last decades, as demonstrated by analyses of prescription patterns of antiandrogens in men diagnosed with localized prostate cancer [7]. On the other hand, the use of ADT both by academic and non-academic urologists has gradually decreased in some countries, such as the USA, perhaps reflecting reimbursement cuts in recent years, or the awareness of potentially serious adverse effects [25].

### Osteoporosis in patients with prostate cancer

#### Mechanisms of bone loss during ADT

**Figure 1:**
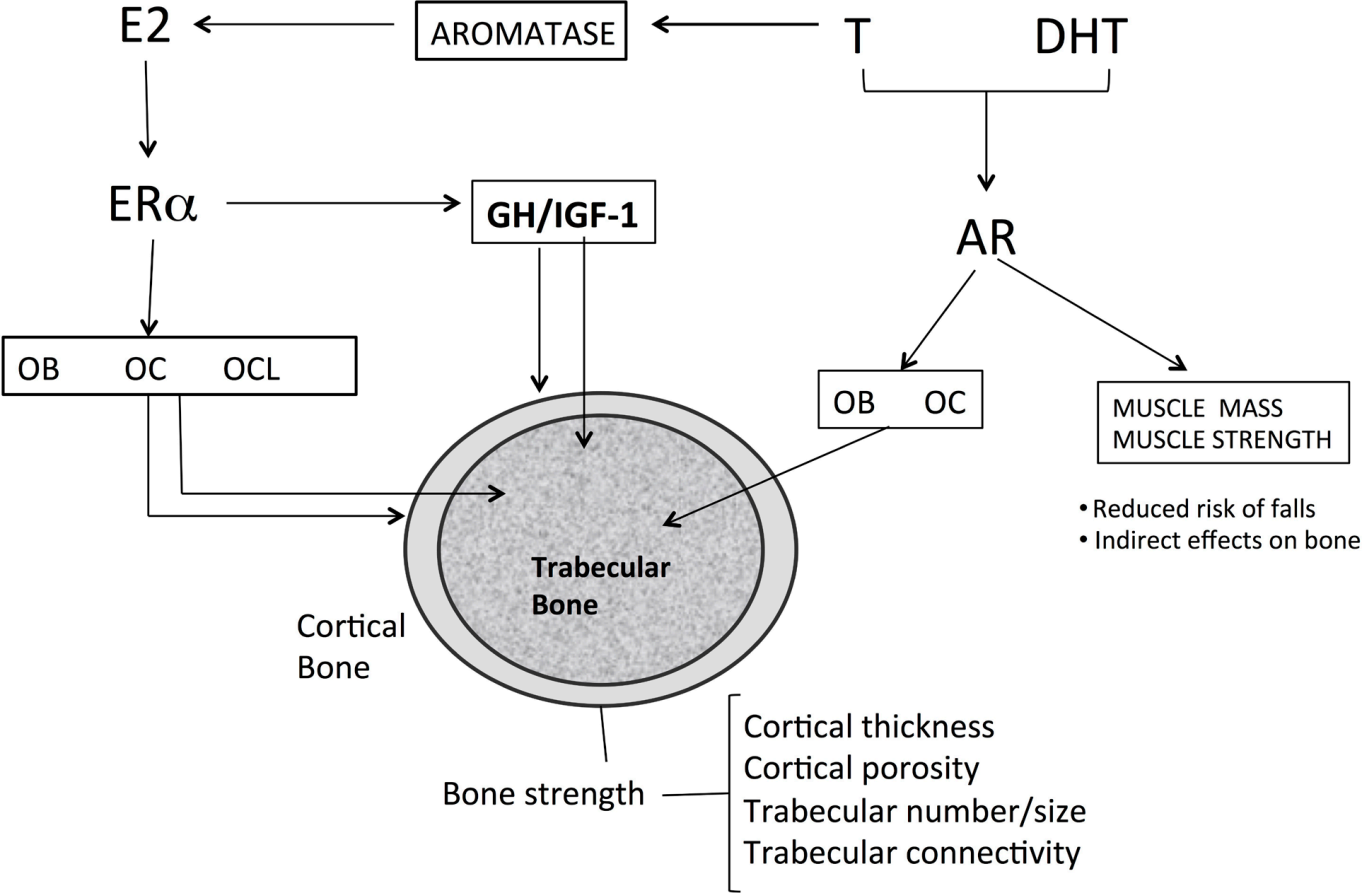
Effects of sex steroids on bone Androgens like T can be converted via aromatization to estrogens and can thus activate both AR and ERα. In males, both AR and ERα maintain cortical and trabecular bone in adult male. Estrogens increases osteoblast number and activity, inhibit osteocyte apoptosis, reduces the number and activity of osteoclasts. Androgen directly increase number and function of osteoblasts and inhibit apoptosis of osteocytes. Osteoclasts apparently do not express AR. Trabecular bone formation is increased by ERα in males, whereas both ERα and AR can inhibit trabecular bone resorption. ERα inhibits endosteal bone resorption and with GH/IGF-1 (probably via central aromatization of androgens) stimulates periosteal bone formation). The action of GH/IGF-1 axis in particularly evident during puberty. E2 : estradiol; T: testosterone; DHT dihydrotestosterone; Era: estrogen a-receptor; AR: androgen receptor; OB: osteoblast; OC osteocyte; OCL :osteoclast; GH: growth hormone; IGF-1: insulin growth-factor;.

ADT in prostate cancer patients reduces serum testosterone levels to the castration range (< 5% of the normal range) and serum estradiol levels to < 20% of the normal level [26]. The importance of sex steroids, mainly estrogen, for the maintenance of bone mass in adult and elderly men has been established by numerous cross-sectional and prospective observational studies showing a strong association between serum levels of total and bioavailable estradiol (E2) with BMD and BMD loss [27–31] (Figure 1). From a molecular point of view, the key mechanism involved is the upregulation of the receptor activator of NF-κB ligand (RANKL) and downregulation of osteoprotegerin induced by estrogen loss, which enhances osteoclast recruitment and activation leading to bone loss [26ì7].

**Figure 2:**
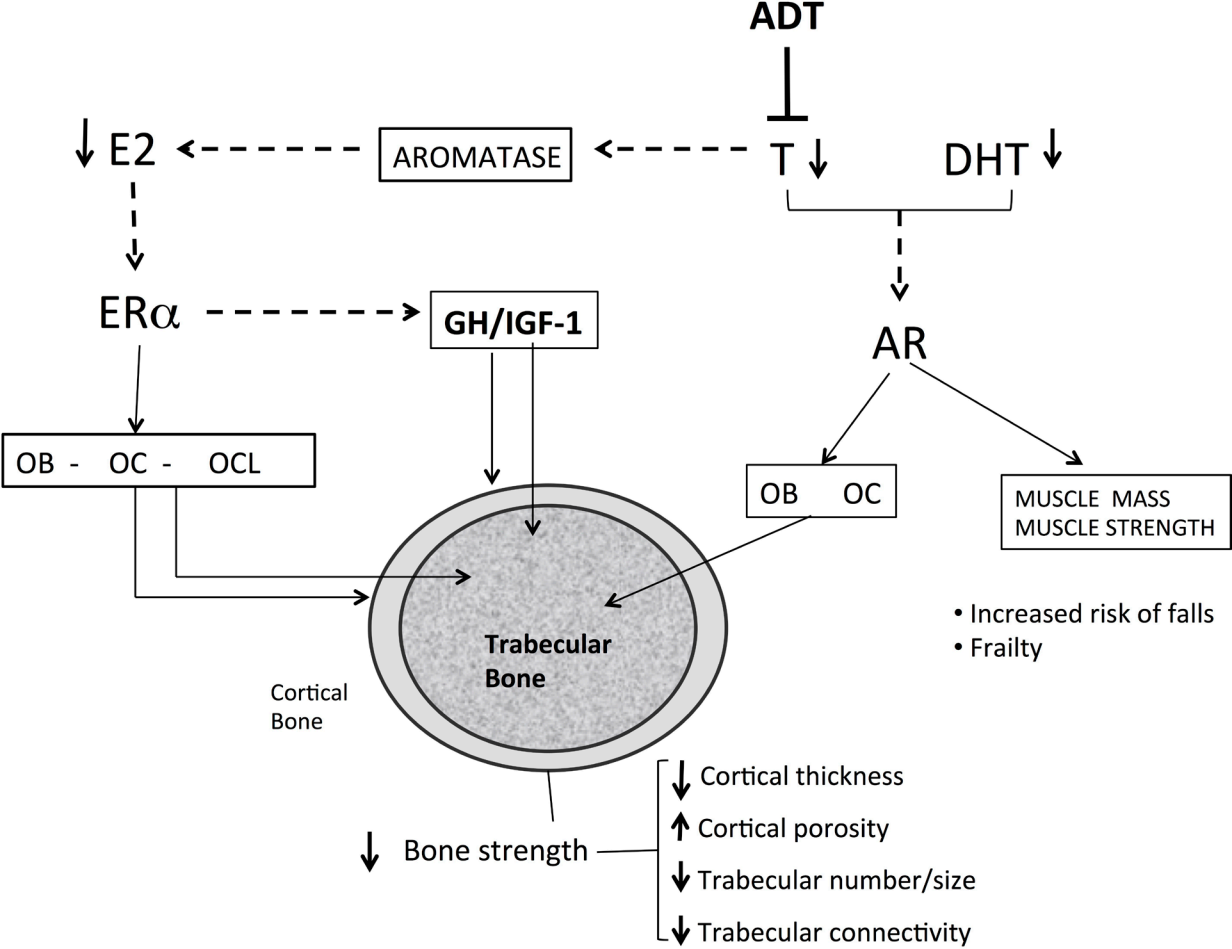
Mechanisms of bone loss in men with prostate cancer receiving androgen deprivation therapy Androgen deprivation therapy reduces testosterone levels and indirectly the estrogen levels in man. The low serum and tissue levels of estrogen increase bone turnover increasing the number of BMU and increasing the number of osteoclasts. The low levels of estrogen increase endosteal reabsorption and cortical porosity. Androgen and estrogen deficiency reduces the thickness and the number of trabeculae and high bone turnover reduce trabecular connectivity, predisposing to bone fragility. Sarcopenia increases risk of falls and indirectly impairs bone metabolism. E2 : estradiol; T: testosterone; DHT dihydrotestosterone; Era: estrogen a-receptor; AR: androgen receptor; OB: osteoblast; OC osteocyte; OCL :osteoclast; GH: growth hormone; IGF-1: insulin growth-factor; ADT: Androgen deprivation therapy; Dotted-line: lack of action

In a cohort of elderly men from Rochester, Minnesota, a threshold for bone loss was found at a bioavailable E2 level of 11 pg/mL (total E2 31 pg/ml) [30]. A similar threshold below which bone loss accelerated at the lumbar spine and femoral neck was reported in other studies [31, 32]. Intervention studies also strongly indicate that estrogen deficiency is the primary mediator of bone loss [33, 34]. Furthermore, low estrogen levels in hypogonadal males are strongly associated, at least in part, in an independent manner, to fracture risk. Several large-scale prospective studies found a relationship between total and bioavailable estrogen levels and fracture incidence with an E2 threshold of 16 and 12 pg/ml, respectively [34, 35]. The contribution of serum testosterone (T) to BMD, bone turnover and fracture risk in hypogonadal men is more complex than that of E2 (Figure 1). Serum T levels have moderate effects on fracture risk and seem to be associated with effects on bone formation, cortical area and extraskeletal factors, such as muscle mass, strength and risk of falls [27]. Sarcopenia and increase in body fat occur as early as 3–6 months after beginning ADT. A mean increase of 7.7% in body fat and about 3% mean reduction in lean body mass have been reported [36]. Bone and skeletal muscle constitute a highly integrated system, and sarcopenia is associated with fractures, not only by increasing the risk of fall, but also by reducing BMD and impairing bone geometry and microstructure [37, 38]. Furthermore, sex steroids may influence local IGF signaling in bone and have an indirect effect on bone via the GH/IGF-1 axis. Low IGF-1 was found to be associated with fracture risk in older men, but no association was found with sex steroids. These relationships should be further investigated [39] (Figure 2).

### Epidemiology of osteoporosis and fragility fractures during ADT

Since ADT is started in almost 50% of men with prostate cancer at some point after diagnosis, and most will take it for at least 2 to 3 years, there is an increasing number of these patients living with severe hypogonadism [40, 19].

Major adverse effects of ADT are consequences of induced hypogonadism that include fatigue, sexual dysfunction, increased fat mass, sarcopenia, and osteoporosis with fragility fractures [41]. It is well known that ADT induces high bone turnover and, consequently, a significantly high rate of bone loss, 4–4.6 % annually, which significantly exceeds that of aging male and postmenopausal women, and is about two-fold that of women with breast cancer treated with aromatase inhibitors [42, 43]. The prevalence of osteoporosis or osteopenia in prostate cancer patients on ADT seems to be very high, with the majority experiencing poor bone health (up to 85%) [44], with variation of prevalence for osteoporosis among the studies (from 9.0 to 53%) influenced by ADT duration, disease stage, ethnicity, and skeletal site of DXA scan [44, 45]. It is noteworthy that the prevalence of osteoporosis in ADT-naive prostate cancer patients is about 4–38%, and men with more advanced disease at diagnosis display a higher prevalence of osteoporosis; therefore, ADT may worsen pre-existent osteoporosis [46–48]. Bone loss seems to be maximal in the first years after the initiation of ADT, ranging from 1.5% to 4.0%, depending on the skeletal site measured [43, 46].

A number of epidemiological studies have associated ADT with an increased risk of fractures. The association between ADT and fractures derived from studies including more than 100.000 men was systematically reviewed, and a summary RR of 1.23 (95% CI, 1.10–1.38) for skeletal fracture and RR 1.39 (95% CI ,1.20–1.60) for vertebral fracture was found [41]. Men with a high baseline risk of skeletal complications develop more fractures after ADT [49]. The fracture rate increased by 19.9 per 1000 person-years (from 52.9 to 73.0 person –years) in men who received 18 or more doses of ADT [49]. As recently demonstrated, patients who received CAB with GnRH agonists and antiandrogens have a higher fracture risk than those who received monotherapy [50]. Mortality after fragility fracture is higher in men, with age-standardized mortality ratios of 2.2–3.2 compared with 1.7–2.2 in women [51]. Men with prostate cancer experiencing a fracture had a 1.38-fold higher overall mortality risk than those who did not (95% CI, 1.34–1.43) [49, 52].

### State-of-the-art in the therapy of osteoporosis in patients affected by prostate cancer

Several RCTs have proven the efficacy of antiresorbing and bone-formative agents commonly employed in men with idiopathic or age-related osteoporosis [53, 54] (Table 1A).

**Table 1:** Randomized placebo-controlled studies demonstrating the efficacy of antiresorptive agents currently approved for the treatment of male osteoporosis (*with the exception of strontium ranelate, which have been approved only in Europe with some restrictions): effect in increasing BMD and preventing fragility fractures in men with osteoporosis and without prostate cancer, and in men with prostate cancer with or without bone metastases

1a. Male osteoporosis						
Study	Treatment period	Patients	Drug tested in the treatment group	Drug regimen	Increase in BMD	Reduction of fracture risk
Orwoll et al. [55]	24 months	n. 241 men with osteoporosis	alendronate	10 mg/day, oral	Yes (spine and hip)	Yes (vertebral)
Boonen et al. [59]	24 months	n. 284 men with osteoporosis	risedronate	35 mg/week, oral	Yes (spine and hip)	No
Boonen et al. [56]	24 months	n. 1199 men with primary or hypogonadal osteoporosis	zoledronate	5 mg/year, i.v.	Yes (spine and hip)	Yes (vertebral)
Orwoll et al. [57] Kaufman et al. [58]	Premature termination (median exposure: 11 months)	n. 437 men with primary or hypogonadal osteoporosis	teriparatide	20 or 40 mcg/day, s.c.	Yes (spine and hip)	Yes (vertebral)
Lyles et al. [60]	24 months	n. 508 men with hip fracture	zoledronate	5 mg/year, i.v.	Yes (spine and hip)	Yes (vertebral and non-vertebral)
Orwoll et al. [61]	12 months	n. 242 with low BMD	denosumab	60 mg/6 months, s.c.	Yes (spine, hip and radius)	No
Kaufman et al. [64]	24 months	n. 261 with osteoporosis	strontium ranelate*	2 g/day, oral	Yes (spine and hip)	No

**1b. Men with prostate cancer without bone metastases (M0) under ADT**						
**Study**	**Treatment period**	**Patients**	**Drug tested in the treatment group**	**Drug regimen**	**Prevention of reduction/increase in BMD**	**Reduction of fracture risk**
Smith et al. [70]	48 weeks	n. 47 men with locally advanced, lymph-node positive or recurrent prostate cancer (M0) starting ADT (leuprolide)	pamidronate	60 mg/12 weeks, i.v.	Yes (spine and hip)	No
Greenspan et al. [80] Greenspan et al. [81]	12–24 months	n. 112 men with prostate cancer (M0) on ADT (GnRH agonists or antiandrogen or combination therapy)	alendronate	70 mg/week, oral	Yes (spine and hip)	No
Klotz et al. [82]	12 months	n. 191 men with prostate cancer (M0) starting ADT (leuprolide acetate)	alendronate	70 mg/week, oral	Yes (spine and hip)	No
Choo et al. [85]	24 months	n. with locally advanced prostate cancer (N0 M0)	risedronate	35 mg/week, oral	Yes (spine and hip)	No
Smith et al. [71]	12 months	n. 106 men with prostate cancer (M0), beginning ADT (GnRH analog with or without antiandrogen)	zoledronate	4 mg/3 months, i.v.	Yes (spine and hip)	No
Israeli et al. [73]	48 weeks	n. 215 men with locally advanced prostate cancer (M0) on ADT (LHRH agonist with or without antiandrogen) for < 12 months	zoledronate	4 mg/3 months i.v.	Yes (spine and hip)	No
Kapoor et al. [75]	12 months	n. 41 men with prostate cancer (M0) on ADT (LHRH agonist or orchidectomy) for < 12 months	zoledronate	4 mg/3 months i.v.	Yes (spine and hip)	No
Bhoopalam et al. [74]	12 months	n. 93 men with prostate cancer (M0) on or starting ADT (LHRH agonist with or without antiandrogen or orchiectomy)	zoledronate	4 mg/3 months i.v.	Yes (spine and hip)	No
Michaelson et al. [76]	12 months	n. 40 men with prostate cancer (M0) receiving GnRH analogs and with T-score > –2.5	zoledronate	4 mg/year i.v.	Yes (spine and hip)	No
Casey et al. [77]	24 months	n. 200 men with prostate cancer (M0) starting ADT (< 30 days of goserelin acetate)	zoledronate	4 mg/3 months i.v.	Yes (spine and hip)	No
Denham et al. [78]	36 months	n. 1071 men with locally advanced prostate cancer (M0) starting ADT (leuprorelin)	zoledronate	4 mg/3 months i.v.	Yes (spine and hip)	No
Smith et al. [88]	36 months	n. 1468 men with prostate cancer (M0) on ADT (GnRH agonist or orchiectomy)	denosumab	60 mg/6 months s.c.	Yes (spine and hip)	Yes (vertebral)

**1c. Men with prostate cancer with bone metastases (M1) under ADT**						
**Study**	**Treatment period**	**Patients**	**Drug tested in the treatment group**	**Drug regimen**	**Increase in BMD**	**Reduction of fracture risk**
Diamond et al. [68]	6 months	n. 21 with metastatic prostate cancer (M1) on ADT (goserelin with antiandrogen for > 6 months)	pamidronate	90 mg single dose i.v.	Yes (spine and hip)	No
Ryan et al. [72]	12 months	n. 42 with non-metastatic and metastatic prostate cancer (M0 and M1) on ADT (orchidectomy or LHRH agonist for < 12 months)	zoledronate	4 mg/3 months i.v.	Yes (spine and hip)	No
Satoh et al. [69]	12 months	n. 40 men with metastatic prostate cancer (M1) starting ADT (GnRH agonist)	zoledronate	4 mg/year i.v.	Yes (spine and hip)	No

In men without prostate cancer, these therapies lead to a net increase in BMD, but there is little evidence for their efficacy in decreasing fracture risk [53]. In men, alendronate, zoledronic acid, and teriparatide have been shown to decrease the occurrence of new vertebral fractures [54–58], while for risedronate no significant effect on fractures was observed, likely due to the small number of fractures [59]. In males after a hip fracture, i.v. zoledronic acid 5 mg once a year has been shown to be effective in reducing the risk of further fractures while improving survival [60]. In males with a BMD T-score between -2 and -3.5 SD or with previous fragility fractures and BMD T-score between -1 and -3.5 (ADAMO trial), the fully human monoclonal antibody against receptor activator of nuclear factor-kB ligand (RANKL), i.e. denosumab 60 mg, administered subcutaneously every 6 months, significantly increased lumbar spine and total hip BMD (by 5.7% and 2.4%, respectively at one year) by directly inhibiting bone resorption, with changes in BMD independent of testosterone levels [61]. Although no data on fracture prevention in men with idiopathic osteoporosis are available, denosumab has been confirmed to be effective and safe in significantly increasing BMD at trabecular and cortical sites [62].

Recently, a meta-analysis has compared the effects on BMD at the lumbar spine and the fracture rate in men among different antiosteoporotic compounds, not including denosumab [63]. The greatest positive change in BMD was observed for zoledronic acid, while teriparatide was ranked first for reducing fracture rate [63].

Alendronate, risedronate, zoledronic acid, teriparatide and denosumab are currently approved for the treatment of male osteoporosis, while strontium ranelate is approved only in Europe, with some restrictions [64] (Table 1A).

Besides the anabolic teriparatide, which cannot be recommended for patients with prostate cancer at risk for osseous metastases, bisphosphonates and denosumab can be employed for the treatment of secondary osteoporosis in men due to ADT. The fact that these antiresorptive drugs have also been proposed to prevent or delay cancer-induced bone disease (i.e. to prevent skeletal related events in CRCP treated with long-term ADT), mainly for their ability to render bone microenvironment unsuitable for cancer cell nesting and implantation, has already been the subject of extensive reviews [65, 66]. Herein, the specific efficacy of antiresorptive drugs in increasing BMD and preventing fragility fractures in non-metastatic, hormone-sensitive prostate cancer patients will be presented.

Parenteral and oral bisphosphonates have been shown to be effective in preventing ADT-induced bone, or increasing lumbar spine and hip BMD, although data on the effect on fracture rate are lacking [67] (Table 1B and 1C). The evidence from studies in men with non-metastatic prostate cancer is described below and further detailed in Table 1B.

Pamidronate was the first bisphosphonate to be tested in a RCT as a preventive therapy for ADT-induced bone loss [70]. In this 48-week-long study, 47 men receiving leuprolide (a gonadotropin releasing hormone agonist) for non-metastatic, but locally advanced, lymph node-positive, or recurrent prostate cancer, were randomized to receive pamidronate 60 mg intravenously every 12 weeks. This treatment was proven to be significantly effective in preventing the reduction in BMD at the hip (*p* < 0.005) and lumbar spine (*p* < 0.0001) provoked by ADT therapy, compared to the placebo-treated group. Indeed, trabecular BMD decreased by 8.5% in men receiving leuprolide alone, whereas it did not change in the leuprolide-pamidronate treated group, in which a marked modification of bone turnover markers was also demonstrated [70].

After the FDA approval of zoledronic acid 4 mg for the treatment of hypercalcemia of malignancy, several studies tested this potent amino-bisphosphonate in bone diseases because of its substantial effects on the skeleton. Several RCTs began to examine its potential action in contrasting ADT-induced bone loss (as reviewed in 67). The first randomized trial of this series tested intravenous (i.v.) zoledronic acid (4 mg/3 monthly) against placebo in men with non-metastatic prostate cancer at the start of treatment with ADT [71]. Mean lumbar spine and total hip BMD increased by 7.8% and 3.9%, respectively, after one year of treatment with zoledronic acid, whereas BMD decreased in the group receiving placebo. In addition, the rise in BMD occurred irrespective of the ADT regimen employed [71]. Comparable results were confirmed in similar studies, in which zoledronic acid was also shown to be effective in reducing bone turnover, and well tolerated [72–75], even when administered in a single annual dose [76]. The latter RCTs demonstrated that zoledronic acid is effective in preventing ADT-induced bone loss even when administered less frequently, regardless of baseline BMD and ADT regimen. Few RCTs have demonstrated zoledronic acid efficacy in preserving or increasing BMD when employed for a period of time longer than one year (up to 36 months) [77, 78], or even when started later in the course of ADT (i.e. more than 1 year) [74]. However, even in the study with the longest follow-up, no difference in incident vertebral fractures was found, despite the positive effect on BMD [78].

The optimal regimen for zoledronic acid in patients receiving ADT has yet to be established. Nonetheless, in a prospective open-label study, no difference in the gain in BMD was noted between groups receiving zoledronic acid 4 mg monthly, bi-monthly, tri-monthly or every six months [79].

It is worth noting that in all these trials performed in patients receiving ADT over the last 15 years, zoledronic acid was employed at the 4 mg dose and not at the 5 mg yearly dose, which is approved for the treatment of idiopathic osteoporosis.

In addition to parenteral bisphosphonates, oral bisphosphonates, such as alendronate (at the dose of 70 mg weekly) and risedronate (at a dose of 2.5 mg daily or 35 mg weekly) have been tested in RCTs in men receiving ADT, and were shown to be well tolerated overall and effective in preserving or increasing BMD at trabecular and cortical sites, while decreasing bone turnover markers [80–85].

In a meta-analysis including data on BMD, rate of fractures, and adverse events from 15 RCTs performed in men receiving ADT for prostate cancer, with or without bone metastases, bisphosphonate therapy had a marked effect in preventing osteoporosis, but also fractures (risk ratio 0.39, *p <* 0.00001 and 0.80, *p =* 0.005, respectively) [86]. Therefore, this study confirmed that bisphosphonates are effective in preserving and preventing bone loss and fractures occurring receiving ADT. Moreover, zoledronate appeared to be more effective than the other bisphosphonates in preventing fractures [86].

As demonstrated in a recent retrospective analysis of administrative databases, however, despite the increased knowledge of ADT-mediated bone loss and evidence of the efficacy of bisphosphonates, the prescriptions of these drugs in hypogonadal prostate cancer patients have remained overall low in the last decade, even in those at high risk for fractures [87].

Besides bisphosphonates, the efficacy of the antiresorptive agent denosumab was tested in men receiving ADT even before being assessed in men with idiopathic osteoporosis, after the initial experience in women receiving aromatase inhibitors for hormone-sensitive breast cancer. In the multicenter, double-blind, placebo-controlled study by Smith et al. (HALT study), 1468 men receiving ADT for non-metastatic, hormone-sensitive prostate cancer, the majority of whom (i.e. 77.9%) had T-score <-1.0 and receiving ADT for more than 6 months, were randomized to receive denosumab (60 mg subcutaneously) or placebo every 6 months, for up to 36 months [88]. The change in lumbar spine BMD at 24 months was set as the primary end point, while the change in total hip BMD at 24 and 36 months and the incidence of newly diagnosed vertebral fractures were set as secondary end points. Lumbar spine BMD increased by 5.6% in the denosumab-treated group and decreased by 1% in the placebo-treated group, with a significant 6.6 difference in percentage points between the two groups. Significant changes in BMD at the total hip, femoral neck and distal third of the radius were also observed at 24 months, with a difference in percentage points between groups of 4.8, 3.9 and 5.5, respectively. Positive changes in BMD in the denosumab-treated patients were observed independently of baseline T-score levels and prevalent vertebral fractures. In the group of men receiving ADT receiving denosumab, a striking decrease in the rate of new vertebral fractures was observed, namely a 62% reduction, at 36 months (1.5% in the denosumab-treated versus 3.9% in the placebo-treated, with a relative risk of 0.38, confidence interval 0.19.0.78, *p <* 0.006), with no difference in the rate of adverse events even in the long term [88]. The magnitude of the effect of denosumab treatment on fracture rate is indeed similar to that demonstrated in the FREEDOM trial in women with postmenopausal osteoporosis receiving the same drug regimen [89]. The gain in BMD and the rapid marked reduction in the rate of bone turnover in men receiving ADT was similar to that observed in the FREEDOM and ADAMO trials [88, 89, 61]. In subgroup analyses of subjects participating in the HALT study, no difference was observed in terms of gain in BMD with respect to different baseline variables such as BMI, age, prevalent fractures, BMD and bone turnover markers, and prior duration/regimen of ADT [90].

Several studies on cost-effectiveness of antiresorptive agents in the prevention of osteoporotic fractures have demonstrated that these drugs are cost-effective or even cost-saving when administered to subjects aged 50 years or older with a risk for fractures above a certain threshold, e.g 20% as established by the FRAX algorithm [91]. Unfortunately, only one cost-effectiveness analysis of this kind has been performed, and only for alendronate in men receiving ADT [92]. This study showed that a BMD assessment, followed by proper therapy with alendronate for 5 years in the case of osteoporosis (as defined by T-score), was cost-effective when performed in men aged 70 years, with locally advanced or high-risk localized prostate cancer starting a 2-year course of ADT after radiotherapy. On the other hand, administering alendronate without a BMD is cost-effective when administered to older men with history of fractures or with a history of lower BMD prior to ADT [92].

Recent trials have investigated the efficacy of antiresorptives as a preventive measure for the development of bone metastases in patients with prostate cancer. In the European Zometa Study (ZEUS) performed in 1433 selected patients with non-metastatic, high-risk localized prostate cancer, zoledronate failed to increase bone-metastases-free survival rates [93]. These results were confirmed in the RADAR trial, in which zoledronate did not offer a clear benefit in this regard [78]. By contrast, denosumab has recently been shown to significantly delay, by a median of 4.1 months, the onset of bone metastases in a study involving 1432 subjects with castration-resistant, non-metastatic prostate cancer [94]. It is likely that high-risk patient subgroups would benefit more from antiresorptive therapy in this respect. Indeed, a subgroup analysis has shown that patients with a shorter doubling time of PSA were likely to benefit more from denosumab administration in terms of bone metastases prevention [95]. However, no positive effects on overall survival rates have been observed in the denosumab-treated group. Moreover, a high incidence of osteonecrosis of the jaw was observed in the denosumab-treated group [94]. For these reasons, denosumab has not been approved for the prevention of bone metastases in men with prostate cancer and without bone metastases.

### Fracture risk assessment in prostate cancer patients receiving ADT and preventive measures

Osteoporosis and the risk of fragility fractures should be assessed in all patients with prostate cancer, and, in particular, in those starting or receiving ADT.

**Figure 3:**
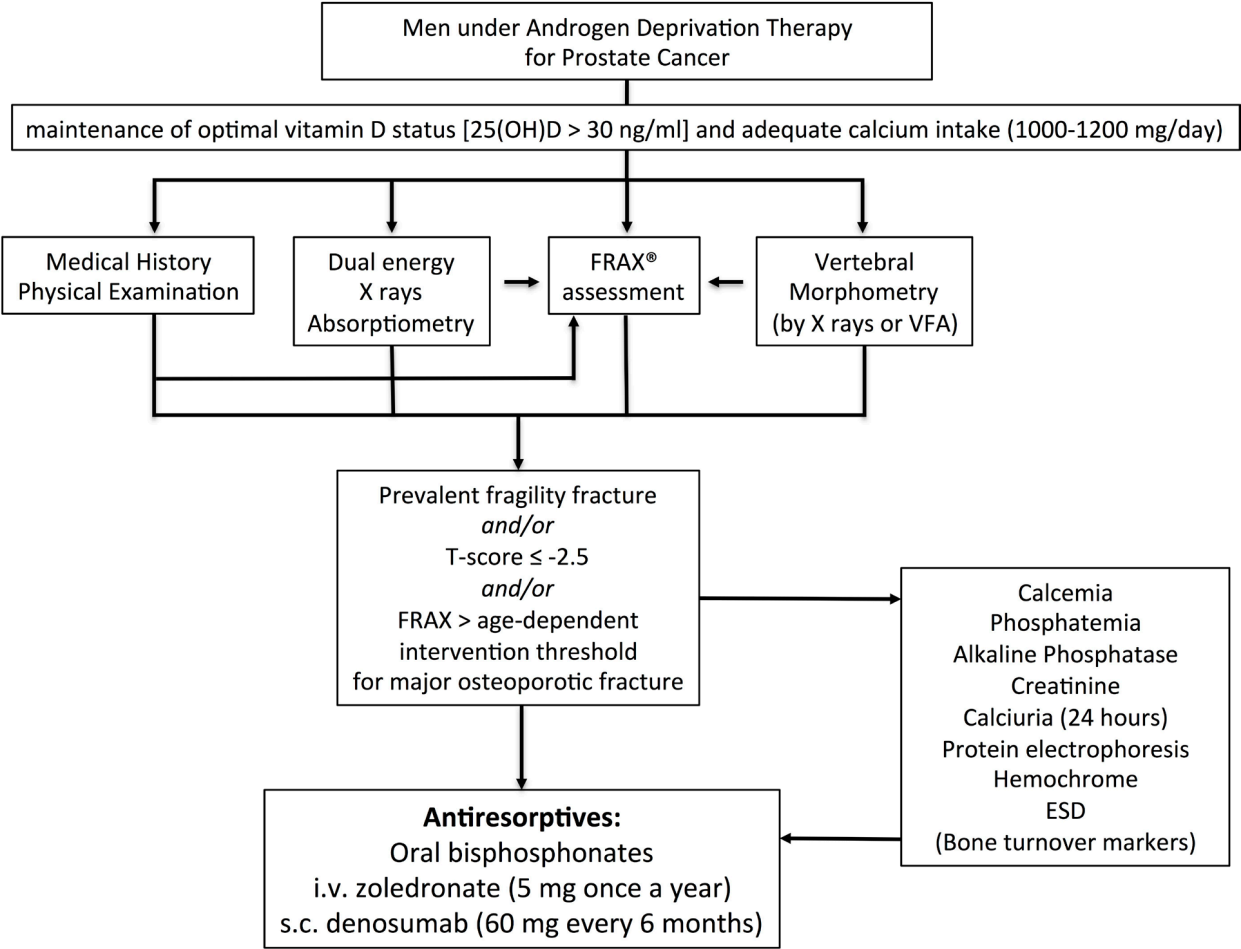
IOF’s algorithm for the management of non-metastatic bone disease in prostate cancer patients receiving ADT (modified from ref. 16)

**Figure 4:**
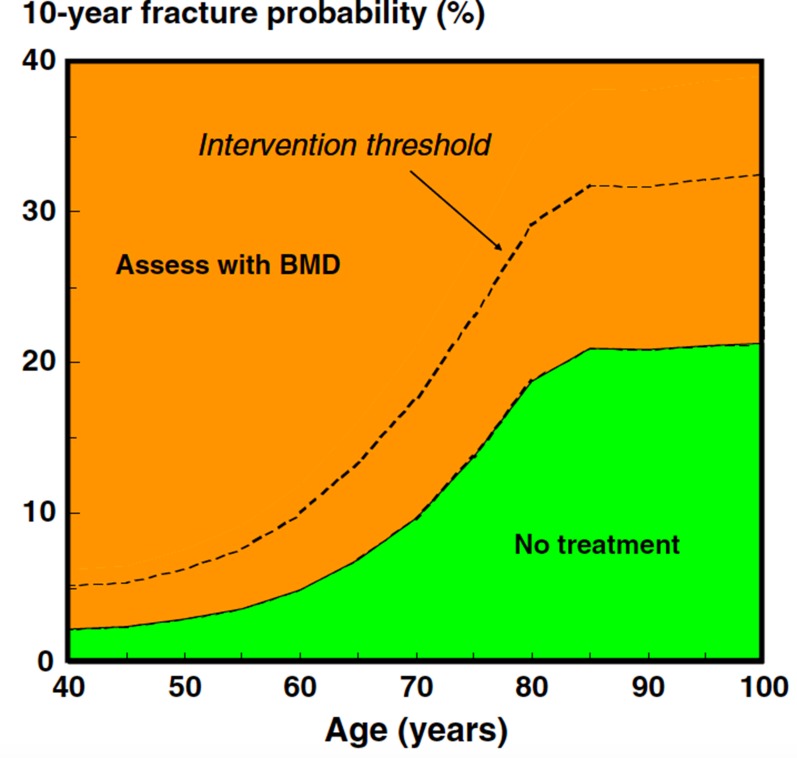
Assessment of fracture risk in countries with high access to DXA: FRAX-based assessment threshold (solid line) and FRAX-based intervention thresholds (dotted line) (reproduced from ref. 100)

After carefully reviewing the medical history and a focused physical examination, the risk for fractures should be estimated by looking at bone mineral density (BMD) by dual energy X-ray absorptiometry (by DXA), and by assessing vertebral prevalent fractures (by X-ray or by DXA) [16] (Figure 3). Low BMD values (i.e. T-score < –2.5 at the total hip, femoral neck or lumbar spine) indicate the need to start appropriate antiresorptive therapy. Since a similar fracture risk has been observed for the same DXA-derived BMD values in men and women, the use of a large Caucasian female referent database for the calculation of T-score in men has been agreed upon [96]. Consequently, a greater proportion of men will experience a fracture at higher T-score values. For this reason, it is necessary not to rely only on the T-score for the selection of men who will receive/take advantage of an antiosteoporotic treatment [97]. In addition to BMD, clinical risk factors have to be taken into account for the diagnosis of osteoporosis. Therefore, the presence of a fragility fracture is sufficient criterion to diagnose osteoporosis, independent of T-score values. In addition to the detection of prevalent fragility fractures, the FRAX algorithm calculated using femoral BMD is a good tool for assessing overall fracture risk, considering “ADT therapy” as secondary osteoporosis [16, 97]. The FRAX computer-based algorithm is a tool that has been specifically developed to estimate fracture risk of patients of both sexes. The FRAX-estimated risk comes from the simultaneous assessment of multiple individual clinical risk factors and may include BMD at the femoral neck for further refinement of the 10-year probability of major osteoporotic fractures or hip fractures [98]. Nonetheless, whether FRAX-derived fracture probability fits ADT risk has not been established; consequently, a FRAX intervention threshold specific for these patients requires validation. Regarding postmenopausal osteoporosis, in the past, some guidelines recommended a 20% intervention threshold for a major osteoporotic fracture and a 3% threshold for a hip fracture. Although these recommendations were based on health economic considerations relevant to the US many years ago, they have been advocated by many authorities, given that they were originally selected by the National Osteoporosis Foundation [99]. Recently, age-dependent thresholds have been validated and adopted for both sexes, first in the United Kingdom [98], then in more than 30 countries, including the European Guidelines [100, 101]. There are noticeable differences in intervention thresholds between countries, because of differing population risks of fracture and death, especially at younger ages. Those recommended for Western Europe are shown in Figure 4.

Recently, the FRAX algorithm has also been demonstrated to predict falls in elderly males [102]. This is of pivotal importance for the evaluation of men receiving ADT, since loss of androgens could also compromise muscle mass and function, therefore increasing the rate of fall and hindering the rehabilitation process after major fragility fractures.

If a diagnosis of osteoporosis is made according to the above criteria, first-line diagnostic tests must always be undertaken in order to exclude secondary causes of osteoporosis (Figure 3). The assessment of bone turnover markers is controversial, although it may be useful for monitoring treatment.

### General recommendations for bone health in patients with non-metastatic prostate cancer

Patients with prostate cancer, and above all those starting or receiving ADT, should have some preventive measures not different from those used for primary prevention of idiopathic osteoporosis (i.e. physical exercise, nutrition, optimal lifestyle, vitamin D supplementation), independent of further bone assessments [16] (Figure 3). It is advisable to test vitamin D status and check daily calcium intake by means of questionnaires in all patients with prostate cancer in order to provide proper supplements and/or appropriate nutrition advice. According to the National Comprehensive Cancer Network’s guidelines on prostate cancer, levels of serum 25(OH)D, a marker for vitamin D status, should be checked and maintained at least above 20 ng/ml (i.e. 50 nmol/L) by means of a daily intake of ≥ 800 IU of cholecalciferol, together with a daily calcium intake of 1000 mg, by means of calcium-rich foods or supplements. The maintenance of a positive calcium balance is mandatory in the case of concomitant therapy with intravenous bisphosphonates or denosumab, because of the risk of hypocalcemia [16].

Several evidence-based practical guidelines for the management of secondary osteoporosis in men with prostate cancer receiving anti-hormonal treatments have been issued [16, 103].

Combining the recommendation from the US-based National Osteoporosis Foundation and Osteoporosis Canada, the IOF experts have agreed that individuals with osteoporosis (T-score a -2.5), and/or with a risk assessed by FRAX exceeding 20% for major fractures, or 3% for hip fractures, and/or displaying vertebral fragility fractures, should be treated with antiresorptives [16] (Figure 3). Nonetheless, IOF experts acknowledge that these indications can be adapted in different regions/countries according to expert guidance and insurance reimbursement policies [16].

Although the optimal regimen and long-term effects of antiresorptives must be established, they are recommended in the above-mentioned conditions, since they are capable of decreasing ADT-mediated bone loss in at-risk individuals. Oral bisphosphonates, such as weekly alendronate or risedronate, zoledronic acid (5 mg) administered once yearly, or denosumab 60 mg dispensed subcutaneously every 6 months, are the treatments of choice. In the selection of antiresorptive therapy, the problem of low adherence to oral bisphosphonates leading to early treatment discontinuation has to be taken into account. For this reason, drugs such as zoledronic acid and denosumab administered parenterally at wider intervals might be preferred.

In high-risk patients placed on antiresorptives, as well as low-risk patients managed conservatively, BMD should be monitored by DXA every 18–24 months. Men receiving ADT with T-score between -1 and -2.5 followed-up without antiresorptive should have BMD measured every 12 months in order to detect small significant changes, particularly at sites rich in cancellous bone (i.e. lumbar spine) placing them in the high-risk category [16].

To date, no evidence-based recommendations can be made on the possible administration of antiresorptives such as denosumab as a preventive measure for bone metastases or pathologic fractures in men receiving ADT for non-metastatic prostate cancer, due to significant side effects and poor evidence of oncologic benefit.

### Research agenda

Several issues need to be addressed in the near future for the maintenance or improvement of bone health in patients treated with ADT for prostate cancer.

The efficacy in terms of BMD gain and fracture risk reduction of emerging therapies targeting skeletal muscle besides bisphosphonates and denosumab needs to be tested in RCTs in men receiving ADT (Table 2).

**Table 2:** Emerging therapies for the treatment of musculoskeletal consequences of androgen deprivation therapy

Agents targeting bone	Agents targeting muscle
Chloride Channel Modulators	Androgen Receptor Modulators (SARM)
Anti-cathepsin K	GH Secretagogues
Anti-Integrins	PPAR-beta Modulators
Src inhibitors	Anti-Myostatin Antibodies
Androgen Receptor Modulators (SARM)	Myostatin Soluble Receptors
GLP2	Anti-Activin II Receptors
Inhibitor of gut serotonin	Angiotensin II Blockades
Anti-sclerostin	Beta-2 Receptor Agonists
Anti-Dickkopf	Anti-IL-6
Modulators of LRPs Pathway	
Anti-activin	Anti-activin

Estrogens have been shown to be important for bone health in men as well as in women. Since estrogens in men derive from the peripheral conversion of testosterone, ADT reduces estrogen levels in parallel to testosterone. Therefore, it is conceivable that increasing their levels in men receiving ADT could be beneficial to bone mass. One study has addressed this issue assessing the effect of a therapy with estradiol on bone density in a small number of men receiving ADT [104]. No significant changes in BMD were observed as a consequence of administration of estradiol alone or in combination with risedronate in men receiving antiandrogens [104]. Nonetheless, the importance of estradiol for male bone mass has raised the question as to whether selective estrogen receptor modulators (SERMs) could increase BMD in men receiving ADT for prostate cancer who develop low estradiol levels. One RCT has recently demonstrated that toremifene, a second-generation SERM, administered at a dose of 80 mg orally daily, is capable of: significantly increasing BMD at the lumbar spine, total hip and femoral neck versus placebo (*p <* 0.0001); decreasing bone turnover markers (*p <* 0.05); and, most importantly, reducing relative risk of new vertebral fractures by 50% versus placebo in a 2 year period [105]. However, in addition to an improvement of lipid profile in the toremifene treated group, there was an increased rate of thromboembolic events with respect to the placebo group (2.6% versus 1.1%, respectively) [105]. Additional studies are therefore needed to better address the value of an estrogen replacement therapy in preserving and preventing bone loss and fragility fractures in men receiving ADT.

Since the loss of muscle mass and function represents a major problem in men receiving ADT, and increases the risk for fall and fractures, physical exercise with specific training programs and therapies addressing skeletal muscle might be advantageous for these patients [67]. Specific therapies targeting skeletal muscle, i.e. the anti-myostatin antibody, have been tested in randomized trials, demonstrating that this treatment is effective in increasing muscle mass but not muscle function [106]. In this respect, selective androgen receptor modulators could be employed to maintain the favorable effects of androgens on muscle and bone while reducing undesirable side effects, as demonstrated by *in vivo* studies in animals [107, 108] and phase II trials in humans for skeletal muscle only [109]. However, RCTs are strongly needed, both in idiopathic osteoporosis and secondary osteoporosis, such as ADT-induced bone and muscle loss, in order to make evidence-based recommendations to decrease the risk of falls.

New therapies developed for the treatment of postmenopausal osteoporosis, such as the cathepsin K inhibitor odanacatib [110] and the antisclerostin antibody romosozumab [111], could be employed to control ADT-mediated bone disease and decrease the risk for fragility fractures in such patients.

In addition to testing new drugs, a refinement of the intervention threshold in men with prostate cancer, which includes consideration of the age of the patients, is strongly advisable, as well as a validation of the FRAX in these subjects, considering competing mortality. Health-economic studies on cost-effectiveness, particularly for denosumab, are needed in order to better inform regulatory agencies in identifying men at high risk for fracture undertaking ADT, for whom treatment is cost-effective or even cost-saving. Additional information on the size of the problem and the budget impact of antiresorptive prescriptions in each country is also required.

On the other hand, as recently demonstrated, significant gaps and barriers remain among prostate cancer specialists regarding the assessment, treatment and monitoring of bone disease in these patients, despite increased knowledge and awareness of the problem [112]. Multidisciplinary units, including bone experts, would be advisable in order to overcome barriers to care for and better identify at-risk subjects. Moreover, further studies assessing the specific impact of different ADT therapies and regimens on bone are needed, to better define and decrease the risk of fragility fractures.

## CONCLUSIONS

Bone disease is a very common complication of ADT in men with hormone-sensitive advanced prostate cancer. The identification of high risk subjects is mandatory in order to select patients for taking advantage of antiresorptives in terms of BMD preservation and possible fracture risk reduction in the long term. The close collaboration of prostate cancer specialists and bone experts is pivotal for the development of shared guidelines for the management and monitoring of non-metastatic skeletal involvement, also taking into account cost-effectiveness analyses.
